# Doppler tissue perfusion measurement is a sensitive and specific tool for a differentiation between malignant and inflammatory pancreatic tumors

**DOI:** 10.1371/journal.pone.0215944

**Published:** 2019-04-29

**Authors:** Przemysław Dyrla, Jerzy Gil, Krzysztof Kosik, Daniel Schneditz, Marek Saracyn, Stanisław Niemczyk, Arkadiusz Lubas

**Affiliations:** 1 Department of Gastroenterology, Military Institute of Medicine, Poland; 2 Institute of Physiology, Medical University of Graz, Graz, Austria; 3 Department of Endocrinology and Isotope Therapy, Military Institute of Medicine, Warsaw, Poland; 4 Department of Internal Medicine, Nephrology and Dialysis, Military Institute of Medicine, Warsaw, Poland; Academy of Sciences of the Czech Republic, CZECH REPUBLIC

## Abstract

Differentiation between pancreatic malignant and inflammatory tumors presents an important diagnostic problem. The ability to recognize pancreatic malignant tumors using Doppler evaluation of tissue perfusion has been recently demonstrated. The aim of the study was to assess the diagnostic value of Dynamic Tissue Perfusion Measurement (DTPM) in the differentiation between malignant and inflammatory pancreatic tumors. The study included 60 patients (35M, 25F, age 60.9 ± 2.3 years) with a malignant (Group 1, n = 30) or inflammatory (Group 2, n = 30) pancreatic tumor undergoing endoscopic ultrasound with the evaluation of tissue perfusion by Color Doppler and a simultaneous biopsy of lesions for cytological evaluation. In 20 patients the diagnosis was verified in the postoperative histopathological examination. Flow velocity (FV) and percentiles of the distribution of perfusion intensity (PR) evaluated by DTPM were analyzed with regard to receiver-operator-characteristics. FV as well as PR were significantly higher in Group 2 compared to Group 1. A threshold of 2.0 cm/sec for FV identified patients with malignancies with a sensitivity of 83% and specificity of 86%. In multivariable regression analysis, the best PR parameter for differentiating between malignant and inflammatory tumors was 97.5% percentile, whose value of 0.922 allowed for the recognition of pancreatic malignant tumors with a sensitivity of 62% and specificity of 83% (p < 0.001). In conclusion, Color Doppler ultrasound tissue perfusion parameters are a sensitive and specific tool in the differentiation between malignant and inflammatory pancreatic tumors.

## Introduction

Differentiation between pancreatic malignant and inflammatory tumors presents an important diagnostic problem. Adenocarcinoma is the most frequent pancreatic malignant tumor accounting for more than 90% of all malignancies of the organ [1,2]. Each year pancreatic cancer is diagnosed in 200,000 new cases (52% men and 48% women). The highest incidence is found in developed countries such as the USA, Australia, Argentina or regions e.g., Central and Northern Europe, and comparable between sexes (8-12/10^5^ male and 6-7/10^5^ female). With a 5-year mortality rate of over 95%, pancreatic cancer is one of the deadliest cancers worldwide. It is the fourth leading cause of cancer-related deaths in both women and men, with an incidence rate similar to the mortality rate [3,4]. Yearly survival in Europe varies between 11.5% and 28.3%, and only 5.7% of patients survive a period of 5 years [5–7]. Therefore, a correct diagnosis of pancreatic focal lesions is a very important diagnostic issue. Endosonography is known to be the best method for diagnosing solid pancreatic lesions [8,9]. When accompanied by endoscopic ultrasound (EUS) fine needle aspiration (EUS-FNA), it is characterized by a high sensitivity and specificity in differentiating pancreatic pathologies [9,10]. Additional administration of contrast agents during that procedure allows to assess the blood flow in a tissue or to observe the changes in echogenicity of a tissue. Contrast-enhanced harmonic EUS (CH-EUS) is used to visualize parenchymal perfusion and microvasculature in the pancreas and plays an important role in the differential diagnosis of conventional EUS-detected solid pancreatic lesions and ductal carcinomas [11,12]. However, this method requires an infusion of ultrasound contrast agent, which rarely can be complicated by adverse events [13]. Contrary to contrast-enhanced ultrasound techniques, in a small group of patients we introduced recently the ability to recognize malignant pancreatic lesions using non-invasive measures of tissue perfusion obtained by Color Doppler examination [14].

The aim of this study was to provide an additional evidence for the diagnostic value of the Dynamic Tissue Perfusion Measurement (DTPM) in the differentiation between malignant and inflammatory pancreatic tumors, in a much larger group of patients.

## Materials and methods

Statutory activity and the consent of the Bioethics Committee of the Military Institute of Medicine was the basis of the present work (project number 245/2014). The research tests were conducted in compliance with the Declaration of Helsinki. All patients provided written informed consent.

From March 2015 to March 2017, consecutive patients presenting to the Department of Gastroenterology with a diagnosed focal pancreatic lesions in the form of carcinoma or chronic pancreatic inflammation, in whom EUS examination using Color Doppler was able to visualize the blood flow within the lesion, were included in the study.

Inclusion criteria: minimum age of 18 years; consent to participate in the study and to perform endoscopic examinations with a biopsy; no contraindications for the EUS examination of the upper digestive tract; solid focal pancreatic lesions observed in EUS examinations; vascularisation within focal pancreatic lesions visible in Color Doppler. Exclusion criteria included: lack of consent to participate in the study and to perform endoscopic examinations with a biopsy; cystic focal pancreatic lesions observed in EUS examinations; lack of possibility to visualize the flow within focal pancreatic lesions in Color Doppler.

EUS including Color Doppler assessment of the perfusion in the pancreatic lesion was performed in all study patients. Patients with malignant pancreatic lesions confirmed in histology/cytology were included into Group 1. All patients with pancreatic inflammatory tumors (Group 2) had a history of acute pancreatitis related to alcohol consumption and/or ductal biliary stones. In ultrasound and CT imaging groove pancreatitis criteria were not found [15,16]. Furthermore, based on the International Association of Pancreatology guidelines, autoimmune pancreatitis was excluded [17,18]. In histological material achieved in EUS-FNA with the use of EchoTip ProCore needles, periductal inflammation and fibrosis with lymphocyte, plasmocyte and macrophage infiltrations, without atypical cells were confirmed.

### Histopathological examination

The material obtained from histopathological and cytological examinations was evaluated in the local pathomorphology department. After receiving the written result, the patient was qualified to Group 1 or Group 2. Patients who were not qualified for surgical treatment were subjected to a 6-month follow-up and re-verification in order to exclude neoplastic process.

### Endoscopic ultrasound and diagnosis

Endoscopic ultrasound (EUS) examination (Pentax EG-3870 UTK, 5–12 MHz linear transducer) was performed with 2D and Color Doppler imaging as well as EUS Fine Needle Aspiration (EUS-FNA) using EchoTip ProCore needles were performed to collect samples for histology/cytology. 20 patients with histopathologically verified lesions (17 adenocarcinomas, 3 inflammatory pancreatic tumors) were qualified for surgery. Lastly, the diagnosis was always confirmed by cytology, postsurgical histopathology, or by a follow-up of at least 6 months in order to exclude malignancy in the patients who did not undergo surgery.

### Perfusion assessment

To assess the perfusion of solid focal pancreatic lesions, during EUS, short (3 to 6 seconds) image sequences were recorded, in which Color Doppler was able to detect the blood flow within the lesion [14]. After the examination, the recorded sequences were transferred and then evaluated by an independent operator using PixelFlux software (Chameleon Software, Leipzig, Germany) and DTPM [19]. A region of interest (ROI) was set in the focal lesion surface area within the Color Doppler frame. The frequency of Color Doppler was set consequently at 5 MHz. We analyzed the changes in the blood flow in at least one vessel within the tumor. Mean flow velocity (FV) within the tumor was evaluated by DTPM. The distribution of perfusion intensity refers, in PixelFlux, to the number of pixels within the ROI that relate to the intensity of the respective value, and is visually expressed as a Perfusion Relief (PR). The value of the perfusion intensity distribution was determined for the 2.5%, 25%, 50%, 75% and 97.5% percentiles (PR2.5, PR25, PR50, PR75 PR97.5) of perfusion intensity.

### Statistics

Differences between perfusion parameters were assessed using *U* Mann-Withney test or Student's t-test depending on the distribution of the data. Chi-square test was used in order to test the differences between nominal variables. Receiver-operator-characteristics (ROC) analysis evaluated the predictive value of perfusion to identify a malignant lesion. Multivariable regression analysis was employed to identify the best parameter in PR differentiating between inflammatory and malignant lesions. Statistical calculations were performed using Statistica 12 software (StatSoft Inc., Cracow, Poland).

## Results

**Fig 1:**
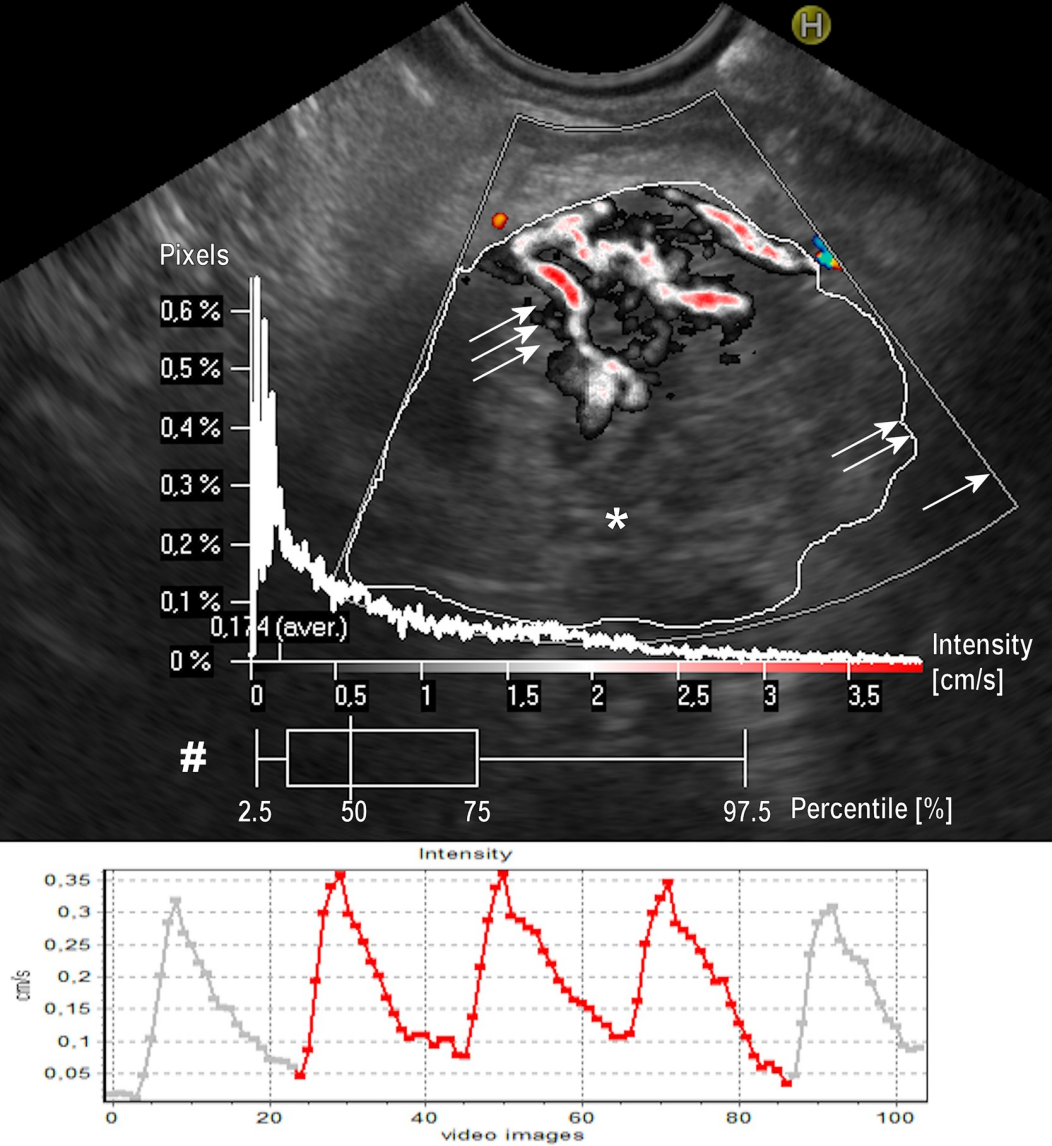
Example of perfusion relief and the flow intensity in a pancreatic inflammatory tumor. Upper part: Graphical presentation of the Perfusion Relief (triple arrow) in the selected ROI (double arrow). The ROI border outlines tumor area (*) within Color Doppler frame (single arrow). A diagram Pixels/Intensity is a quantitative evaluation of PR showing how many pixels within the ROI relate to the intensity of the respective value. A box-plot diagram (#) expresses the distribution of pixel intensity, in percentiles (pc) (whisker: 2.5% and 97.5%; box 25% and 75%; vertical line in the box 50%—median). In the presented case mean intensity in the ROI was 0.174 cm/s, median PR intensity (50% pc) was 0.593 cm/s, whereas 97.5% pc was 2.887 cm/s. Lower part: Graphical presentation of mean temporary values of the flow intensity (average 0.94 cm/s) within selected ROI in the same patient. The Y-axis represents intensity in cm/s. The X-axis represents consecutive images in the video file.

**Fig 2:**
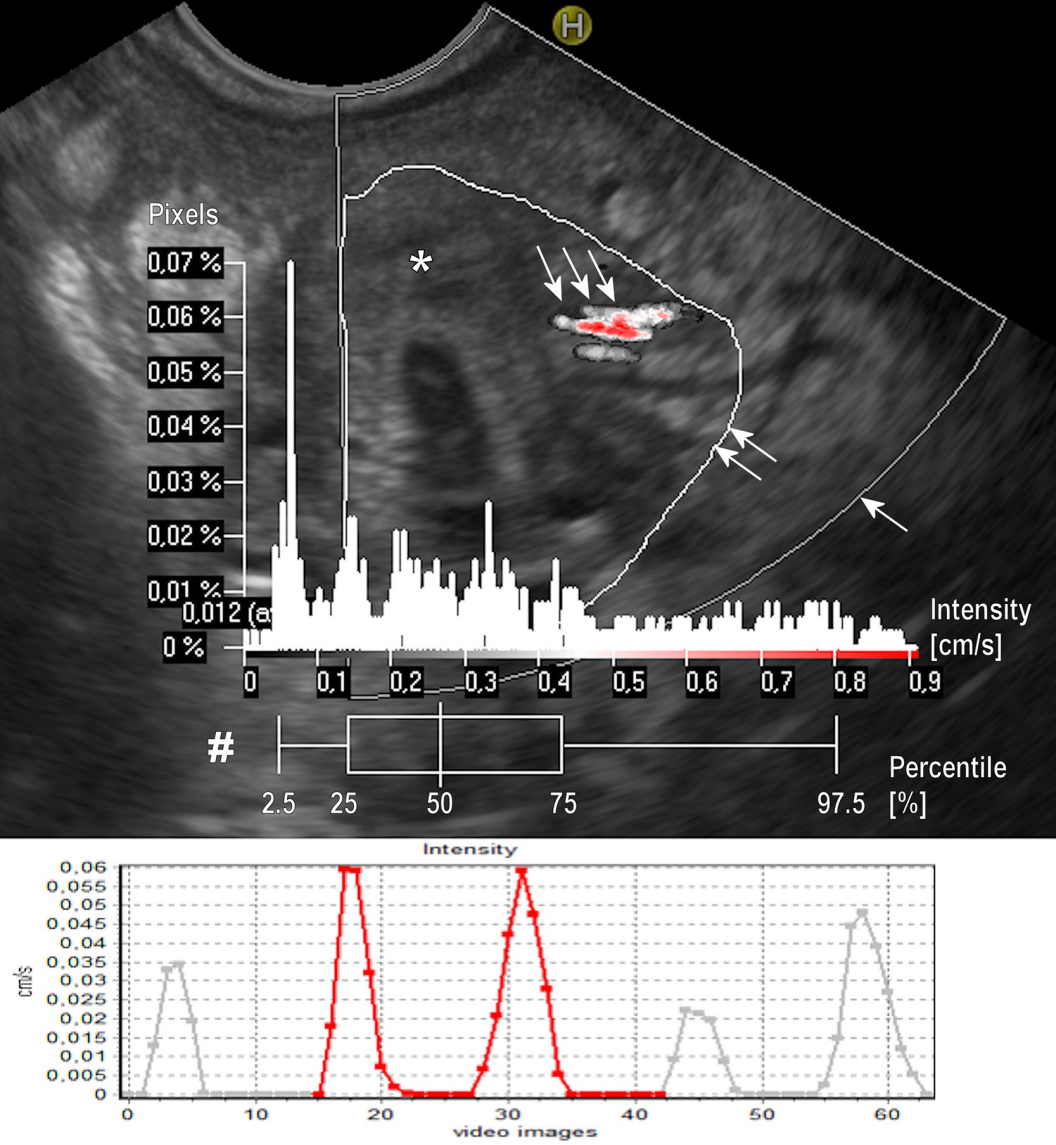
Example of perfusion relief and the flow intensity in a pancreatic malignant tumor. Upper part: Graphical presentation of the Perfusion Relief (triple arrow) in the selected ROI (double arrow). The ROI border outlines tumor area (*) within Color Doppler frame (single arrow). A diagram Pixels/Intensity is a quantitative evaluation of PR showing how many pixels within the ROI relate to the intensity of the respective value. A box-plot diagram (#) expresses the distribution of pixel intensity, in percentiles (pc) (whisker: 2.5% and 97.5%; box 25% and 75%; vertical line in the box 50%—median). In the presented case mean intensity in the ROI was 0.012 cm/s, median PR intensity (50% pc) was 0.267 cm/s, whereas 97.5% pc was 0.802 cm/s. Lower part: Graphical presentation of mean temporary values of the flow intensity (average 0.006 cm/s) within selected ROI in the same patient. The Y-axis represents intensity in cm/s. The X-axis represents consecutive images in the video file.

**Fig 3:**
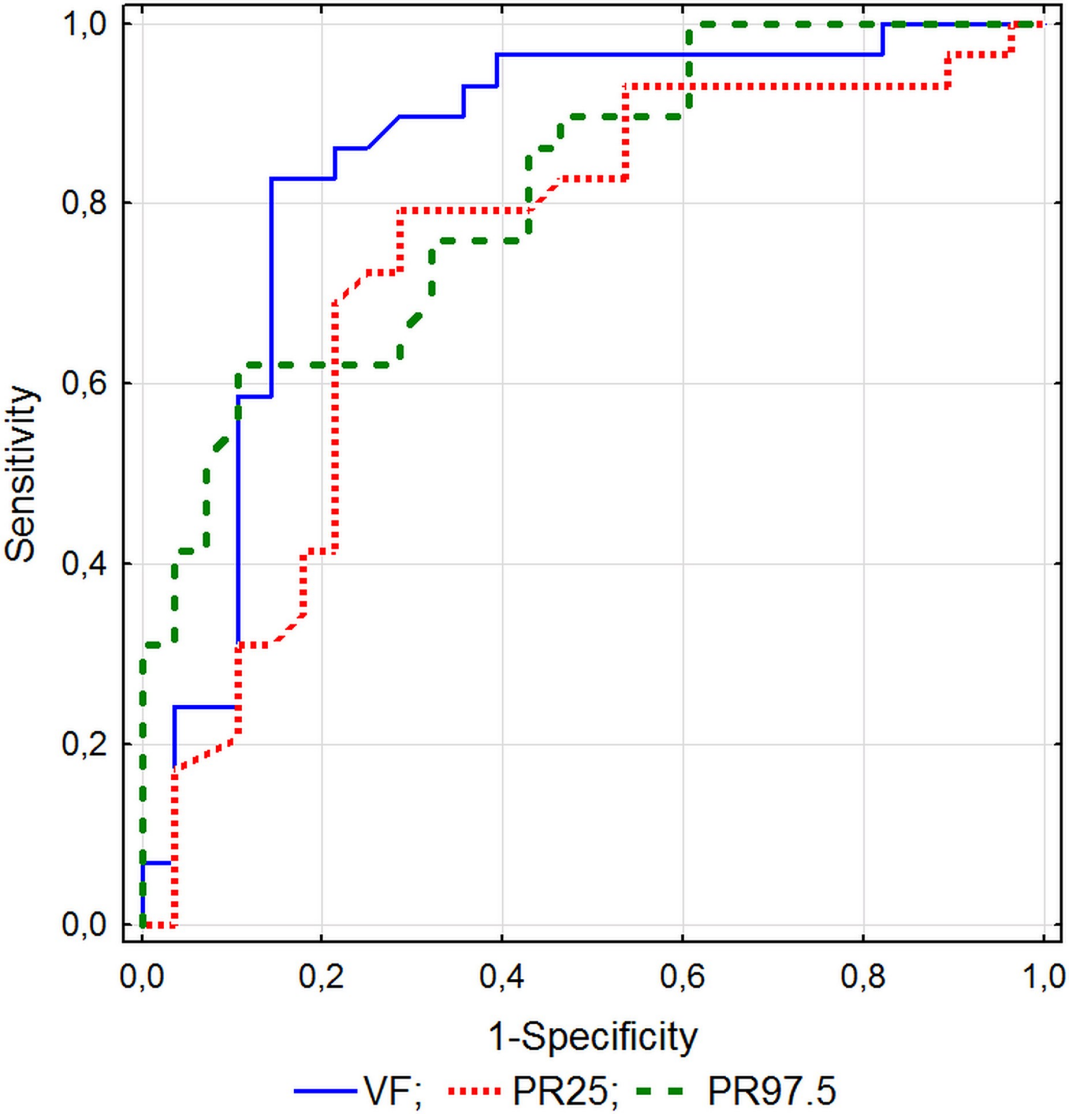
Comparison of ROC curves for Doppler perfusion parameters differentiating between inflammatory and malignant pancreatic tumors.

The study included 60 consecutive patients (35 M, 25 F, age 60.9 ±2.3 years) (S1 Table) presenting to the Department of Gastroenterology with the diagnosed focal pancreatic lesion in the form of carcinoma (Group 1; n = 30) or chronic pancreatic inflammation (Group 2; n = 30). One patient with a malignant tumor and 2 patients with chronic pancreatitis were excluded from evaluation because of invisibility of the flow within the lesion (only peripheral vessels visible). In total, perfusion was examined in 57 patients divided into the following groups: Group 1 (n = 29; 21M, 8F, age 63.1 ±13.0) with diagnosed malignant tumors and Group 2 (n = 28; 14M, 14F, age 56.6 ±11.3) with pancreatic focal inflammatory lesions (Table 1). Baseline characteristics of included patients are presented in Table 2. Groups did not differ in age, BMI, gender structure and presented symptoms. In Group 1, significantly lower mean flow velocity was observed, compared to the group with inflammatory lesions (Table 3, Figs 1 and 2). In the ROC analysis, FV values ≤ 2.0 cm/s identified patients with malignancies with a sensitivity of ≤ 82.8% and specificity of ≥ 85.7% (Table 4). In Group 1, the distribution of perfusion intensity was significantly lower in all but one (PR2.5) considered percentiles compared to Group 2 (Table 3). Multivariable regression analysis was performed in three models, each containing three successive PR percentiles so as to identify the best percentile interval distinguishing both groups. In each model, only the highest percentile was found to be suitable for the differentiation between the groups. Finally, in the model containing three highest percentiles (PR50, PR75, PR97.5), only the PR97.5 value was identified as an independent factor differentiating between malignant and inflammatory pancreatic tumors (R^2^ = 0.26, p < 0.001). The ROC analysis performed for PR97.5 showed that a value of ≤ 0.922 allowed to recognize malignant lesions with a sensitivity of ≤ 62% and specificity ≥ 89% (AUC 0.813; p < 0.001) (Table 4). However, the PR97.5 value of ≤ 1.198 suggested the potential to distinguish pancreatic adenocarcinomas with a sensitivity of ≤ 76% and specificity ≥ 68%. Although the FV predictive value for the differentiation between malignant and inflammatory pancreatic lesions appears to be better than PR97.5, areas under the curve (AUC) of the considered perfusion parameters were not significantly different (0.852 vs 0.813, p = 0.91) (Fig 3).

**Table 1:** Histopathological and cytological diagnoses in considered groups.

Diagnosis	Group 1 (n = 29)	Group 2 (n = 28)
adenocarcinoma	17 (surgery) 12 (EUS-FNA)	
inflammatory tumor		3 (surgery) 25 (EUS-FNA)

EUS-FNA Endoscopic Ultrasound Fine Needle Aspiration

**Table 2:** Baseline characteristics of included patients.

	All (n = 57)	Group 1 (n = 29)	Group 2 (n = 28)	p—Value
Age (y)	60.42 ±12.36	62.69 ±13.11	58.07 ±11.29	0.160
BMI (kg/m^2^)	23.40 ± 3.06	23.49 ± 2.44	23.32 ± 3.64	0.367
Male/Female	35/22	21/8	14/14	0.082
Weight loss	13	6	7	0.698
Abdominal pain	25	12	13	0.701
Jaundice	12	7	5	0.561
Other*	12	8	4	0.218

* Other symptoms: irregular bowel movements, diarrheas, tympanites, renal/hepatic colic etc.

For nominal variables (weight loss, abdominal pain, jaundice, other) sum of positive results of the examination is presented.

**Table 3:** Results of DTPM in pancreatic tumors in considered groups.

	Group 1 (n = 29)	Group 2 (n = 28)	p—Value
**FV (cm/s)**	1.584 ±0.646	2.653 ±0.813	< 0.001
PR2.5*	0.008 (0.004; 0.028)	0.011 (0.003; 0.035)	0.067
PR25*	0.039 (0.017; 0.316)	0.071 (0.012; 0.447)	0.001
PR50*	0.118 (0.038; 0.702)	0.252 (0.032; 1.658)	< 0.001
PR75*	0.255 (0.075; 1.431)	0.656 (0.075; 3.676)	< 0.001
PR97.5*	0.796 (0.186; 2.309)	1.802 (0.553; 5.723)	< 0.001

FV—flow velocity; PR2.5, PR25, PR50, PR75, PR97.5 –percentiles of the distribution of perfusion intensity, respectively 2.5%, 25%, 50%, 75%, 97.5%

*—median and range (min; max) presented.

**Table 4:** ROC data of DTPM parameters in differentiating malignant and inflammatory pancreatic tumors.

Variable	OCP	Sensitivity (%)	Specificity (%)	PPV	NPV	ACC	AUC	ERR
**FV (cm/s)**	2.009	82.8	85.7	0.857	0.828	0.842	0.852	0.158
	2.303*	89.7	71.4	0.765	0.870	0.807	0.852	0.193
**PR25**	0.062	79.3	71.4	0.742	0.769	0.754	0.741	0.264
	0.057*	75.9	71.4	0.733	0.741	0.737	0.741	0.263
**PR97.5**	0.922	62.1	89.3	0.857	0.694	0.754	0.813	0.246
	1.198	75.9	67.9	0.710	0.731	0.719	0.813	0.281

ACC–accuracy; AUC–area under curve; ERR–error rate; FV—flow velocity; NPV—negative predictive value; OCP–optimal cut-off point; PPV–positive predictive value; PR25, PR97.5,–percentiles of the distribution of perfusion intensity, respectively 25%, 97.5%

*—value approximated to previous data [14].

## Discussion

In the present work, the first attempt was made to determine the usefulness of DTPM parameters in the differentiation between malignant and inflammatory pancreatic tumors. A new method based on the ultrasound Color Doppler results is DTPM which allows for quantitative and semi-automatic evaluation of perfusion in the study area. Quantification of the Color Doppler flow signal by the original software is the basis for DTPM. It was originally intended to evaluate the perfusion parameters of renal parenchyma, and then successfully employed in neurology, gynecology, oncology, gastroenterology (intestinal perfusion), and more recently in the diagnosis of cardio-renal disturbances [19–22]. Thus far, contrast-enhanced ultrasound (CEUS) has been used to assess the blood perfusion in focal pancreatic lesions and to differentiate between inflammatory lesions and pancreatic cancer [23]. The administration of intravenous contrast agent in ultrasound examination enables the calculation of the mean transit time through the tissue, using the curves of changes in echogenicity of the tissue. Two values are calculated: the time required to restore a full strengthening of the tissue and the intensity of the strengthening. The product of these two values is a measure of the flow rate. Contrast-enhanced harmonic EUS shows that the majority of pancreatic cancers exhibits hypovascular heterogeneous enhancement because of irregular network-like microvessels. The analysis of perfusion using contrast enables to differentiate poorly vascularized lesions of pancreatic cancer from focal lesions observed in autoimmune pancreatitis. [24,25]. The differentiation between perfusion of pancreatic focal lesions was verified in Contrast Enhanced Computed Tomography (CE-CT). Kandel et al. found that perfusion in pancreatic cancer was significantly lower than the perfusion in normal pancreatic tissue [26]. Perfusion in 64-slice CE-CT was used by Lu et al. to distinguish between pancreatic cancers and inflammatory tumors [27]. In this study, perfusion imaging blood volume was lower by 53% in cancers compared to inflammatory tumors. Similar conclusions were obtained by Klauss et al., who applied dynamic sequences in 64-slice CE-CT in 25 patients with diagnosed pancreatic cancer [28]. Morphology of perfusion curves in CE-CT was useful in the evaluation of pathological pancreatic lesions. In the study by Zamboni et al., CE-CT allowed for the diagnosis of pancreatic cancer with a sensitivity of 74%, specificity of 94%, positive predictive value of 92%, and negative predictive value of 79% [29]. The analysis of the perfusion in focal pancreatic lesions was also performed by magnetic resonance imaging [30]. In pancreatic cancers, diffusion parameters were significantly lower which allowed for the proper diagnosis. The above-mentioned data confirm our observation of a much reduced FV in malignant tumors compared to inflammatory ones. Moreover, percentiles of the distribution of perfusion intensity were significantly lower in malignant lesions compared to non-malignant ones. We recently demonstrated the ability to adequately differentiate malignant pancreatic lesions from inflammatory and benign cystic lesions using DTPM method on the small group of patients [14]. In this study, both reduced FV and PR25 significantly differentiated pancreatic malignant tumors from inflammatory and cystic lesions. However, cystic tumors can already be diagnosed with a high accuracy already in the EUS study, based on the identification of anechoic areas confined by smooth walls. In addition, due to the small number of patients, the PR25 parameter was chosen arbitrarily, as a representative of the perfusion relief parameters. In the present work, we confirmed our previous observation and the value of DTPM for differentiating between malignant and inflammatory pancreatic lesions in a representative group of patients. Again, FV was significantly lower in malignant tumors compared to inflammatory ones, and a threshold of ≤ 2.0 cm/sec allowed to recognize malignant lesions with a sensitivity and specificity of approx. 84%. In contrast to our previous study PR97.5 was the best factor differentiating between malignant and inflammatory pancreatic tumors. A PR97.5 of ≤ 0.922 recognized malignant pancreatic lesions with a sensitivity of 62% and specificity of 89%. Our study provides evidence for the feasibility of perfusion parameters to differentiate between malignant and inflammatory pancreatic tumors. EUS using Doppler evaluation of tissue perfusion is also invasive because of combining endoscopy with FNA but does not require the administration of contrast agent. The diagnostic properties of FV and PR97.5 in recognizing pancreatic malignancies, obtained in our study, can be estimated as good [31]. Despite the promising results, our study has several limitations. Although the evaluation of perfusion in a pancreatic tumor based on the flow detected in only a single vessel can be misleading with contributory poor imaging conditions, it is only possible to record a reduced, not increased flows. Thus, overdiagnosis of malignant lesions seems to be a better option than omitting them. The literature provides sufficient evidence that endosonography is the best method for detecting solid pancreatic lesions. Nevertheless, it is still difficult to differentiate between malignant and benign lesions [6–8]. This is one of the first studies showing the features and benefits of ultrasound evaluation to differentiate solid pancreatic lesions, which can be considered as equivalent to CH-EUS or even CT [14]. This method can be successfully used in the appropriate group of pancreatic tumors and can help in the diagnosis of malignant lesions. Despite the promising results of non-invasive imaging methods the true nature of the lesion must be confirmed by histology.

## Conclusions

Color Doppler ultrasound-derived tissue perfusion measurement is a sensitive and specific method for the differentiation between pancreatic malignant and inflammatory tumors. Measures of the flow velocity and parameters of perfusion distribution demonstrate comparable diagnostic properties. A comparative analysis of Doppler perfusion methods with other already used diagnostic tools allowing to detect pancreatic malignant tumors should be performed.

## Supporting information

S1 Table Results of DTPM in pancreatic tumors. (XLSX)
